# A Tetraspecific VHH-Based Neutralizing Antibody Modifies Disease Outcome in Three Animal Models of Clostridium difficile Infection

**DOI:** 10.1128/CVI.00730-15

**Published:** 2016-09-06

**Authors:** Diane J. Schmidt, Gillian Beamer, Jacqueline M. Tremblay, Jennifer A. Steele, Hyeun Bum Kim, Yaunkai Wang, Michele Debatis, Xingmin Sun, Elena A. Kashentseva, Igor P. Dmitriev, David T. Curiel, Charles B. Shoemaker, Saul Tzipori

**Affiliations:** a Department of Infectious Disease and Global Health, Cummings School of Veterinary Medicine, Tufts University, North Grafton, Massachusetts, USA; b Department of Animal Resources Science, Dankook University, Cheonan, Republic of Korea; c School of Agriculture and Biology, Shanghai Jiao Tong University, Shanghai Key Laboratory of Veterinary Biotechnology, Key Laboratory of Urban Agriculture (South) Ministry of Agriculture, Shanghai, People's Republic of China; d Department of Radiation Oncology, Washington University, St. Louis, Missouri, USA; University of Florida

## Abstract

Clostridium difficile infection (CDI), a leading cause of nosocomial infection, is a serious disease in North America, Europe, and Asia. CDI varies greatly from asymptomatic carriage to life-threatening diarrhea, toxic megacolon, and toxemia. The incidence of community-acquired infection has increased due to the emergence of hypervirulent antibiotic-resistant strains. These new strains contribute to the frequent occurrence of disease relapse, complicating treatment, increasing hospital stays, and increasing morbidity and mortality among patients. Therefore, it is critical to develop new therapeutic approaches that bypass the development of antimicrobial resistance and avoid disruption of gut microflora. Here, we describe the construction of a single heteromultimeric VHH-based neutralizing agent (VNA) that targets the two primary virulence factors of Clostridium difficile, toxins A (TcdA) and B (TcdB). Designated VNA2-Tcd, this agent has subnanomolar toxin neutralization potencies for both C. difficile toxins in cell assays. When given systemically by parenteral administration, VNA2-Tcd protected against CDI in gnotobiotic piglets and mice and to a lesser extent in hamsters. Protection from CDI was also observed in gnotobiotic piglets treated by gene therapy with an adenovirus that promoted the expression of VNA2-Tcd.

## INTRODUCTION

Clostridium difficile infection (CDI) is currently one of the leading causes of nosocomial infection (1, 2) and is fast becoming a cause of community-acquired diarrhea in previously low-risk populations, including children, healthy adults, and pregnant women (1–7). Manifestations of CDI vary from asymptomatic colonization; mild or moderate diarrhea; a severe or fulminant illness with complications, including pseudomembranous colitis, toxic megacolon, and small bowel ileus; or even systemic inflammatory response syndrome, a multisystem organ failure that can be fatal (8). The emergence of antibiotic-resistant hypervirulent strains and the increase in disease relapse have complicated the treatment of CDI, leading to increases in hospital stay, morbidity, and mortality (1).

Clostridium difficile is a Gram-positive, spore-forming anaerobic bacterium that produces two toxins, designated TcdA and TcdB (9), which are the major virulence factors of CDI (10). They are large exotoxins that bind to human colonocytes, causing inflammation, fluid accumulation, and mucosal injury manifested as pseudomembranous colitis (11).

C. difficile survives, persists, and produces the two exotoxins in the gut after prolonged treatment with broad-spectrum antibiotics reduces normal microflora (12). The extensive use of antibiotics for treatment of CDI has increased the emergence of resistant strains, leading to a dramatic increase in the incidence of disease relapse estimated at 20% to 35% (13). Consequently, there is an urgent need to develop novel, nonantibiotic therapies that prevent persistence and toxin production by C. difficile and minimally impact normal gut microflora. Ideally, approaches that specifically target toxins instead of bacterial cells and eliminate the possibility of antimicrobial resistance are favored (14, 15). Several therapeutic approaches are currently under development, including antibiotics (8, 16, 17), probiotics (18–23), fecal transplants (24–26), toxin-binding resins or polymers (27), vaccines (16, 28–30), and toxin-specific antibodies (Abs) (31–38). Several but not all antitoxin antibodies improve CDI outcomes in animal models and clinical trials (32, 34, 35, 39–42), but these conventional antibodies are costly and challenging to engineer. There is some evidence from the pig model (43) that antibodies against TcdB alone may be sufficient for treating CDI; however, there are conflicting data on the roles of the toxins in disease (44–46).

As an efficient alternative, we produced and tested heavy-chain-only V_H_ domains (VHHs), generated by Camelidae species, seeking VHHs that neutralize each of the two C. difficile toxins. DNAs encoding these unconventional IgGs (IgG2 and IgG3) are easily cloned (47) and can be expressed at high levels in soluble form (48). The VHH protein products are generally more stable than conventional antibodies and frequently bind the active sites of targeted proteins (48–50). We previously showed that bispecific VHH-based neutralizing agents (VNAs) are highly efficacious as antitoxins in animal models of exposures to botulinum neurotoxins (51), ricin (52), Shiga toxins (53), and anthrax (54), significantly outperforming their monomer VHH components. To achieve protection from CDI, a VNA was engineered and expressed in bacteria containing four VHHs, two (AH3, AA6) that neutralize TcdA and two copies of the 5D VHH (5D, 5D) that neutralizes TcdB (41). This VNA, called ABA, provided potent protection from CDI in a mouse model.

While some reports have indicated that TcdA does not play a significant role in disease pathogenesis in the gnotobiotic pig model of CDI (43), other evidence has shown that TcdA and TcdB toxins contribute to fulminant disease in hamsters (55) and in some mouse models of CDI (56). Since VHH agents remain functional when linked into multimers, we have chosen to include VHHs that neutralize both Tcd toxins in our antitoxin agent, as this should be effective in all of the models of CDI. In the current study, we chose to reengineer the ABA VNA based on recent results (57) and unpublished data showing that two different toxin-neutralizing VHHs against the same target combined into a single linked construct create a more effective antitoxin *in vivo* than a homodimer of only one toxin-neutralizing VHH. In our new VNA, VNA2-Tcd, we replaced one of the two copies of the 5D VHH in ABA with a different TcdB-neutralizing VHH, E3. Specifically, VNA2-Tcd is a tetraspecific agent that contains 5D and E3 VHHs targeting TcdB linked to the two TcdA-neutralizing VHHs, AH3 and AA6. In this report, we test the ability of VNA2-Tcd to protect against CDI pathology in mouse, hamster, and gnotobiotic piglet models of this disease when administered as a protein therapeutic or by adenoviral gene therapy.

## MATERIALS AND METHODS

### Ethics with IACUC statement.

Treatment and care of all animals used in experiments followed institutional animal care and use committee guidelines. All animal studies performed were approved by the Tufts University Institutional Animal Care and Use Committee.

### Plasmid construction.

Synthetic DNA was prepared in which the coding DNAs for the two most potent neutralizing VHHs that we reported previously for TcdA (AH3, AA6) and TcdB (5D, E3) (41) were linked together, each separated by DNA encoding a flexible spacer (GGGGS)_3_, to encode a VHH tetraspecific, heteromultimeric VNA (AH3/5D/E3/AA6) called VNA2-Tcd. This DNA was inserted into expression vector pET32b in fusion with Escherichia coli thioredoxin as described by Tremblay et al. (53) to create the Trx/VNA2-Tcd expression plasmid.

### Protein purification.

The Trx-VNA2-Tcd-6His/pET32b plasmid was transformed into Rosetta-gami (DE3) E. coli, and fermentation was as follows: 20 liters of LB medium with 100 μg/ml ampicillin and 34 μg/ml chloramphenicol was incubated at 15°C and expression induced with 1 mM isopropyl-β-1-thiogalactopyranoside (IPTG) at an optical density at 600 nm (OD_600_) of 0.6 for 20 h (58). The protein was captured by Ni affinity chromatography and eluted with a 0.5 M imidazole gradient at pH 7.5. It was further purified by gel filtration chromatography (HiLoad Superdex 200 26/60; GE Life Sciences) with 20 mM HEPES (pH 7.8), 200 mM NaCl, 1 mM dithiothreitol (DTT), and 1 mM EDTA elution buffer. The protein eluted as a monomer from the gel filtration column. Recombinant protein was visualized by SDS-PAGE/Coomassie and by Western blotting using an anti-E-tag antibody (Bethyl) at a 1:5,000 dilution. In some experiments, the protein was treated for endotoxin removal using the Triton X114 phase partitioning method. The final endotoxin concentration in the endotoxin-free preparation was below 0.01 endotoxin units (EU)/mg as determined by the PyroGene recombinant Factor C assay (Lonza). Fermentation, purification, dialysis, and endotoxin removal were performed by ARVYS Proteins Inc. (Trumbull, CT).

### Adenovirus vector construction and preparation.

The generation of recombinant replication-incompetent adenovirus type 5 (Ad5)-based vectors has been previously described (59). Briefly, in a modification from reference 60, the pShCMV-JGf7 shuttle plasmid was used for subcloning the VNA2-Tcd coding sequence (54) under the control of the mammalian cytomegalovirus (CMV) promoter and was followed by the bovine growth hormone poly(A) signal. A control vector Ad/VNA1-Stx was created in a similar manner with the sequence from two VHHs against Shiga toxins (Stx) (61). This control vector results in the secretion of a Stx-reactive Ab with no binding to Tcd. Each shuttle plasmid was linearized and employed for homologous recombination with the pAdEasy-1 plasmid carrying the viral genome, resulting in the selection of the plasmid containing the recombinant Ad5 genome that incorporates either the VNA2-Tcd or VNA1-Stx expression cassette in place of deleted viral E1 genes. The resultant plasmids were validated by PCR, restriction analyses, and sequencing to confirm the incorporation of the VNA2-Tcd or VNA1-Stx expression cassette within the corresponding recombinant viral genome. The plasmids were linearized with PacI to release the inverted terminal repeats of the viral genomic DNA and transfected into 293 cells to rescue replication-incompetent Ad/VNA2-Tcd. The rescued Ad/VNA2-Tcd and Ad/VNA1-Stx vectors were upscaled, purified by centrifugation on CsCl gradients as previously described (60), and dialyzed against phosphate-buffered saline (PBS) (8 mM Na_2_HPO_4_, 2 mM KH_2_PO_4_ [pH 7.4], 137 mM NaCl, 2.7 mM KCl) containing 10% glycerol and stored at −80°C. The titers of physical viral particles (vp) were determined by the methods of Maizel et al. (62).

### Neutralization assay.

Vero cells (ATCC) at a concentration of 2.4 × 10^4^ cells/100 μl of medium (Dulbecco modified Eagle medium [DMEM] high glucose plus 1 mM sodium pyruvate, 2 mM l-glutamine, 50 U/ml and 50 μg/ml penicillin [Pen]/streptomycin [Strep] [pH 7.4] [HyClone]) were plated in 96-well plates overnight for 90% to 95% confluence, prior to the addition of VNA2-Tcd added in serial dilutions (in medium) from 100 μg/ml to 1.0 fg/ml and either 2 ng to 12.5 ng/ml TcdA and 0.25 to 2 ng/ml TcdB or TcdA and TcdB in a 24-h cytotoxicity/cell rounding assay (41).

### Enzyme-linked immunosorbent assay.

EIA/RIA 96-well high-binding plates (Corning Costar) coated with 0.5 to 5 μg/ml of recombinant TcdA (rTcdA) or rTcdB or rTcdA + rTcdB overnight at 4°C were used for binding assays. Plates were washed 3 times with 1× PBS + 0.1% Tween followed by 3 times with 1× PBS and blocking solution (4% to 5% nonfat dry milk in 1× PBS + 0.1% Tween) for 1 h at room temperature (RT) with rocking. Serially diluted VNA2-Tcd, serum, or fecal samples diluted in blocking solution were incubated for 1 h at RT with rocking and were washed as above. Equivalent control samples were spiked with a known amount of VNA2-Tcd for use as an internal standard. Goat anti-E-tag horseradish peroxidase (HRP)-conjugated antibody (Bethyl Labs), diluted 1:5,000 in blocking solution, was incubated for 1 h at RT with rocking, washed as above before adding 3,3′,5,5′-tetramethylbenzidine (TMB) microwell peroxidase substrate (KPL) to develop (incubated for 10 to 40 min), stopped with 1 M H_2_SO_4_, and read at 450 nm on a ELx808 ultra microplate reader (BioTek Instruments) (51). VNA2-Tcd levels in unknown samples were determined by comparison of their signals to those of internal standards as previously described (60, 61, 63–65).

### Mouse systemic toxin challenge.

Six-week-old C57BL/6 female mice were intraperitoneally (i.p.) injected with a single dose of VNA2-Tcd (50 μg/mouse) 1 h prior to i.p. injection of TcdA (100 ng/mouse), TcdB (200 ng/mouse), or TcdA plus TcdB (100 ng/mouse and 200 ng/mouse, respectively). Mice were monitored for signs and symptoms of toxemia (including lethargy, depression, anorexia, dehydration, ruffled coat, and hunched posture). Moribund mice were euthanized following IACUC-approved removal criteria.

### Mouse CDI challenge.

To mimic the human condition and facilitate colonization with C. difficile, ten 6-week-old C57BL/6 female mice received filter-sterilized antibiotics (kanamycin, gentamicin, colistin, metronidazole, and vancomycin) in drinking water for 5 days followed by 2 days of water alone. After 2 days of drinking water, each mouse received one 100-μl i.p. injection of clindamycin (2 mg/ml). One day later, mice were orally challenged (66) with 10^6^ spores of an NAPI/027/BI C. difficile strain, which was designated strain UK6 (67) only (control group), or inoculated with spores and administered VNA2-Tcd (25 to 50 μg/mouse) at 4, 24, and 48 h postchallenge (treated group). Blood was collected at 72, 96, and 120 h postchallenge for VNA titers.

### Hamster CDI challenge.

Again, to mimic the human condition and facilitate colonization with C. difficile, seventeen 110- to 135-g male golden Syrian hamsters were administered clindamycin (30 mg/kg of body weight) via oral gavage for 5 days prior to oral inoculation with 1,000 C. difficile strain UK6 spores. Infected control hamsters were administered clindamycin inoculated with UK6 spores and given sterile PBS i.p. 2 times per day for the duration of the experiment. VNA2-Tcd-treated hamsters were administered clindamycin, inoculated with spores, and given purified VNA2-Tcd (1 mg/kg) i.p. 2 times a day for the duration of the experiment. A blood sample was collected at time of euthanasia for detection of VNA2-Tcd in serum. Necropsies were performed on euthanized animals, and tissues were collected for histopathologic examination.

### Pig CDI challenge.

Thirty gnotobiotic piglets were derived via Caesarean section and maintained in sterile isolators for the duration of the experiment (65). Five groups of piglets were orally inoculated with 10^6^ C. difficile UK6 spores (groups 1 to 5), and group 6 was the uninfected control group (summarized in Table 1). Group 1 (*n* = 3) received VNA2-Tcd (1 mg/pig) 4 h prior to oral inoculation with spores, and group 2 (*n* = 3) received VNA2-Tcd (1 mg/pig) 18 h after oral inoculation with spores. After the initial dose, the treated groups received 2 doses of VNA2-Tcd (1 mg/pig) per day either i.p. or intramuscularly (i.m.) for the duration of the experiment (Table 1). The Ad/VNA2-Tcd-treated group (group 3; *n* = 9) was given 1.0 × 10^11^ viral particles intravenously (i.v.) 1 day prior to oral inoculation with 10^6^ C. difficile UK6 spores and 3 days postinfection (Table 1). Group 4 (*n* = 6) received VNA-Tcd buffer as a control 4 h prior to oral inoculation with 10^6^ C. difficile UK6 spores and at 24 h postinoculation and then every 12 h until the termination of the experiment (Table 1). Group 5 was given control adenovirus expressing an unrelated VNA (*n* = 6) at 1.0 × 10^11^ viral particles i.v. 1 day prior to oral inoculation with 10^6^ C. difficile UK6 spores and 3 days postinfection (Table 1). Group 6 (*n* = 3) was uninfected (Table 1). From all piglets, fecal samples were collected for bacterial culture and blood samples were collected 1 to 3 times (when possible) during the experiment and at the time of euthanasia for VNA titers. Necropsies were performed on all animals, and tissues were collected for histopathologic examination.

**TABLE 1:** Pig treatment groups

Group	No. of pigs	Control buffer (ml)	Control adenovirus (vp)	VNA2-Tcd (mg)	Ad/VNA2-Tcd (vp)	Time (h)	Route
1	3	NA^*a*^	NA	1	NA	−4, 24, then every 12	i.p.
2	3	NA	NA	1	NA	18, then every 12	i.m.
3	9	NA	NA	NA	1.0 × 10^11^	−24, 72	i.v.
4	6	1	NA	NA	NA	−4, 24, then every 12	i.m.
5	6	NA	1.0 × 10^11^	NA	NA	−24, 72	i.v.
6	3	NA	NA	NA	NA	NA	NA

a NA, not applicable.

### Histology.

Tissue samples were collected during necropsy and preserved in 10% neutral buffered formalin. Formalin-fixed samples were embedded in paraffin, sectioned at 5 μm, and stained with hematoxylin and eosin using routine histochemical techniques at Tufts University Cummings School of Veterinary Medicine (TCSVM) Histopathology Service Laboratory (http://vet.tufts.edu/histology-service/). Light microscopic examination and lesion evaluation were performed by a board-certified veterinary pathologist (G. Beamer) with results reported as previously described for severity (minimal, mild, moderate, marked), epithelial ulceration, luminal contents, and quantification (64). Briefly, a quantitative assessment of colitis severity was performed by counting neutrophilic foci in colon sections from each sample. Foci were observed between colonic crypts in the lamina propria in 10 random fields with ×20 magnification.

## RESULTS

### VNA2-Tcd construction, expression, and protein purification.

**FIG 1:**
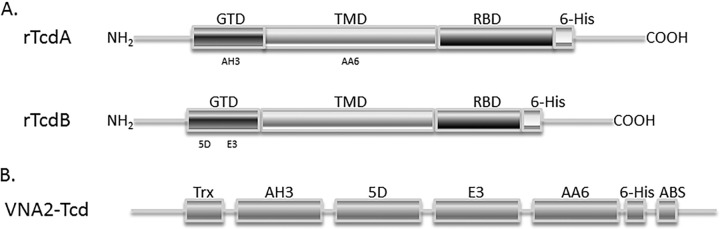
Synthesis and purification of VNA2-Tcd. (A) Schematic diagram of TcdA and TcdB toxins, respectively. GTD, enzymatic glucosyltransferase domain; TMD, transmembrane domain; RBD, receptor binding domain. AH3 and AA6 bind in the GTD region and the TMD region of TcdA, respectively, and 5D and E3 bind to different regions of the GTD in TcdB. (B) Diagram of the heterotetramer VNA2-Tcd, which was synthetically generated and contains two potent neutralizing VHHs to each toxin. The protein contains a thioredoxin protein at the amino end, followed by four VHHs separated by a flexible spacer peptide with a carboxyl-terminal E-tag peptide and an albumin-binding peptide to increase serum persistence.

We previously identified two individual VHHs with strong neutralizing activity against TcdA (AH3, AA6) and two VHHs that neutralized TcdB (5D, E3). Results with these VHHs, and VHHs to other toxins (51–53), have shown that covalently linking two toxin-neutralizing VHHs into bispecific heterodimer VHH-based neutralizing agents (VNAs) results in substantially enhanced *in vitro* and *in vivo* antitoxin potency compared to equimolar pools of unlinked VHHs. We thus engineered the recombinant expression of a tetraspecific VHH heteromultimer containing the four different VHHs, two each having TcdA- and TcdB-neutralizing properties, which were all separated by flexible spacer peptides (Fig. 1). The VNA2-Tcd heterotetramer was purified using Ni affinity and gel filtration chromatography, treated for endotoxin removal using a detergent-based method (>0.01 EU/mg), and visualized following SDS-PAGE, revealing a purity of about 71% for full-length heterotetramers, and the remaining protein bands were almost entirely represented by trimeric and dimeric truncations of full-sized VNAs based on Western blotting (see Fig. S1 in supplemental material). For gene therapy, an adenovirus vector (Ad5) was engineered to promote the expression and secretion of VNA2-Tcd (Ad/VNA2-Tcd) following transduction of mammalian cells.

### *In vitro* characterization.

**FIG 2:**
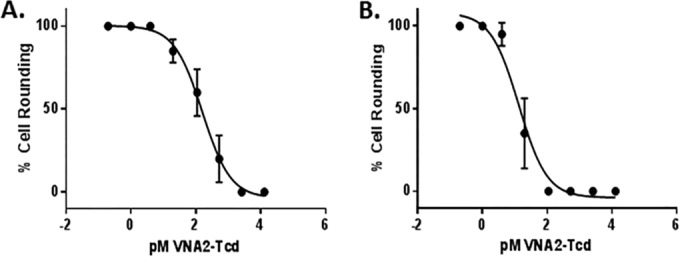
*In vitro* and *in vivo* neutralization of toxins. (A and B) *In vitro* neutralization assay using Vero cells incubated with 100 pM TcdA or 0.6 pM TcdB per well for 24 h with serial dilutions of VNA2-Tcd as indicated. Percent cell rounding (cytotoxicity) was assessed after 24 h. The IC_50_ was determined using the GraphPad nonlinear fit of log-transformed concentrations. Log IC_50_ for TcdA = 2.0 and the IC_50_ was 106.4; Log IC_50_ for TcdB = 1.24 and the IC_50_ was 13.3.

The 50% inhibitory concentration (IC_50_) of VNA2-Tcd was determined by dilution cytotoxicity assay using Vero cells and 100 pM of recombinant TcdA or 0.6 pM of recombinant TcdB and serially diluted VNA2-Tcd. The estimated IC_50_ was about 100 pM for TcdA and about 10 pM for TcdB, indicating that VNA2-Tcd is capable of neutralizing both toxins when present at near equimolar doses to the two Tcd toxins in these assays (Fig. 2A and B).

### Mouse systemic toxin challenge.

We assessed the potency of VNA2-Tcd to neutralize toxins A and B in a systemic mouse challenge using 6-week-old C57BL/6 female mice. Six groups of mice (5 mice per group) were injected i.p. with TcdA and/or TcdB in the presence or absence of VNA or VNA alone and were monitored for signs of toxemia. Initial systemic toxin doses of 100 to 200 ng of TcdA and 100 to 800 ng of TcdB were tested individually to determine the 100% lethal dose (LD_100_) at 24 h. The LD_100_ doses for each toxin were then used in the systemic challenge. The control group mice that were administered 100 ng/mouse of TcdA and 200 ng/mouse of TcdB (i.p.) were either moribund (euthanized) or died within 4 h postchallenge. Other groups were administered the following treatments: (i) no treatment, (ii) VNA2-Tcd only (no toxin), (iii) VNA2-Tcd + TcdA, (iv) VNA2-Tcd + TcdB, or (v) VNA2-Tcd + TcdA/TcdB. VNA2-Tcd treatments were a single i.p. injection of VNA2-Tcd (2.5 mg/kg) 1 h prior to toxin challenge. No mice in these 5 groups (see Table S1 in the supplemental material) showed any signs or symptoms of toxemia, and all remained healthy until termination of the experiment at 7 days post-toxin challenge.

### Mouse CDI challenge.

**FIG 3:**
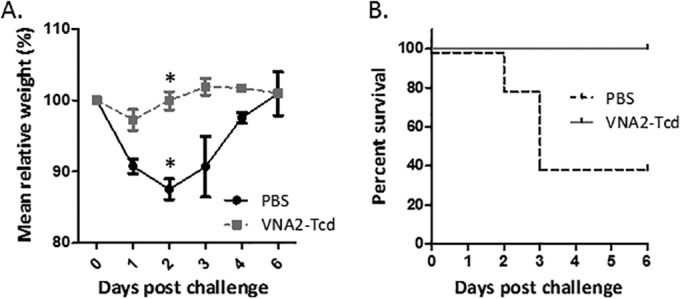
Protection against CDI in mice using VNA2-Tcd. Mice were treated with an antibiotic cocktail for 3 days in drinking water and given a single injection of clindamycin i.p. 1 day prior to infection with 10^6^ PFU of Clostridium difficile UK6 spores alone or UK6 spores and 3 doses of VNA2-Tcd (2.5 mg/kg at 4, 24, and 48 h) postinfection. (A) Weight of mice in treated (VNA2-Tcd) and untreated (PBS) groups during the 6-day study. A Mann-Whitney U test was performed to compare the control and VNA-treated mice per day. Only day 2 (*) showed statistically significant *P* values of 0.021 weights between the control and treated groups. (B) Survival percentage with time for each group.

Two groups (5 mice each) of 6-week-old C57BL/6 female mice were treated for 5 days with an antibiotic cocktail in drinking water and administered a single i.p. injection of clindamycin prior to being orally challenged with 10^6^ C. difficile (UK6 strain) spores. The control group received spores plus PBS, and the treated group received spores and VNA2-Tcd injected i.p. at 4, 24, and 48 h postchallenge. The VNA2-Tcd group received an initial dose of 25 μg/mouse (1.25 mg/kg) at 4 h postchallenge and two additional doses of VNA2-Tcd (50 μg/mouse [2.5 mg/kg]) at 24 and 48 h postchallenge. The control group (spores + PBS) experienced weight loss at days 1, 2, and 3 postchallenge, and most mice began to gain weight on day 3 or 4 postchallenge, while the VNA2-Tcd-treated group experienced weight loss only on day 1 postchallenge and began to regain weight on day 2 postchallenge (Fig. 3A). The difference in weight between the control and treated groups was only statistically significant on day 2 postchallenge. One hundred percent of the control animals developed diarrhea, and 60% were moribund or died (Fig. 3B). Only 1 mouse in the VNA2-Tcd-treated group developed diarrhea, which was resolved by day 2 postchallenge. All mice in the VNA2-Tcd-treated group survived for the duration of the experiment (Fig. 3B). Blood was collected from mice 24, 48, or 72 h after administration of the final dose of VNA2-Tcd. Serum VNA2-Tcd levels were measured by enzyme-linked immunosorbent assay (ELISA) and ranged from 1.9 to 6.1 μg/ml (see Fig. S2A in the supplemental material). Serum TcdA and TcdB neutralization abilities tested by cytotoxicity/cell rounding assay were similar for all treated mice with a 1:10 dilution of serum providing about 50% protection from rounding (see Fig. S2B).

### Hamster CDI challenge.

**FIG 4:**
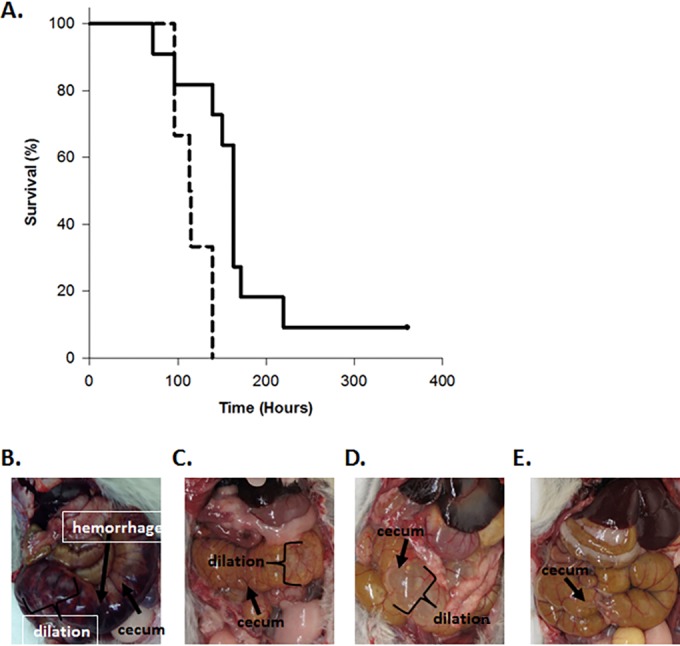
Protection against CDI in hamsters using VNA2-Tcd. (A) Survival percentage with time for hamsters treated with VNA2-Tcd. The *x* axis shows the hours after oral challenge with 1,000 UK6 spores. The *y* axis shows the percentage of survival. The solid black line indicates survival in the VNA2-Tcd-treated group, and the dashed black line indicates survival in the control (PBS) group. The grouped survival data were analyzed by applying a Kaplan-Meier log rank test using SigmaPlot (version 13.0; Systat Software, Inc.). The log rank statistic for the survival curves is greater than would be expected by chance, as the two curves show a statistically significant difference (*P* = 0.003). (B) Cecum (arrowhead) of a control hamster showing hemorrhage (arrow) and dilation. (C) Cecum (arrowhead) of a control hamster showing dilation. (D) Cecum (arrowhead) of a VNA2-Tcd-treated hamster showing dilation. (E) Cecum of treated hamster showing no dilation.

Hamsters were reported to be relatively resistant to the UK6 strain of C. difficile (68), so we performed initial experiments and established that treating with 5 days of oral clindamycin followed by 1,000 UK6 spores induced CDI disease in 100% of Syrian hamsters (not shown). To test VNA2-Tcd efficacy, two groups of male golden Syrian hamsters were administered clindamycin (30 mg/kg) orally for 5 days prior to inoculation with 1,000 UK6 spores. The control group (6 hamsters) received spores plus PBS, and the treated group (11 hamsters) received spores and 1 mg/kg of VNA2-Tcd injected i.p. two times a day for the duration of the experiment. All hamsters in the control and treated groups except for one treated hamster developed diarrhea. In the control group, hamsters developed diarrhea between days 5 through 7, and all were moribund and euthanized by day 7 (Fig. 4A). As expected for the CDI hamster model, control hamsters (6/6) developed mild to severe cecal dilation with or without hemorrhage (Fig. 4B and C). The hamsters treated with purified VNA2-Tcd also developed diarrhea between day 4 and day 13 (10/11), but there was a trend for less severe cecal dilation and hemorrhage (Fig. 4D and E) and a delay in the onset of symptoms (not shown). By Kaplan-Meier log rank test, survival was significantly increased by VNA treatment (Fig. 4A; *P* = 0.003). Overall, the time frame suggests that the main beneficial effect was delayed morbidity, as 90% of all hamsters eventually succumbed by 200 h (8 days) post-spore administration. In the mouse model, untreated mice succumbed to infection by day 2 or 3 post-spore inoculation, and none of the treated mice became moribund.

Blood was collected from hamsters at the time of euthanasia, and VNA2-Tcd serum levels were measured by ELISA, ranging from 500 to 25,000 ng/ml (see Fig. S3A in the supplemental material). Since blood was collected at the time of euthanasia only, which occurred at various times throughout the experiment, and hamsters received twice daily injections of VNA2-Tcd i.p., the levels of VNA in serum varied widely, likely due to how soon after the last treatment hamsters became moribund.

No significant differences were detected by light microscopic examination of large intestinal tissue samples, including the cecum, from control and treated groups (see Table S2 and Fig. S3B in the supplemental material). Together, the clinical observations, necropsy, and microscopic findings suggest that morbidity in the hamster CDI model includes disease mechanisms that are independent from those resulting in edema and neutrophilic inflammation. Possibilities include electrolyte imbalances secondary to diarrhea and dehydration, hypovolemic shock due to fluid loss, and poor perfusion/reduced venous return to the heart secondary to compression of the caudal vena cava from massively dilated caeca.

### Pig CDI challenge treated with the purified VNA2-Tcd protein.

**FIG 5:**
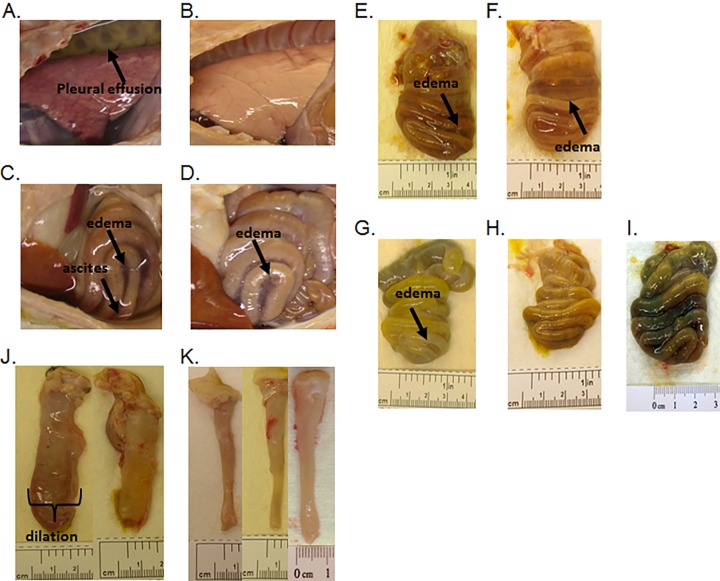
Necropsy images from piglets infected with C. difficile. (A) Control piglet with pleural effusion (arrow), lung displaying diffuse hyperemia and congestion. (B) VNA2-Tcd-treated piglet with a normally aerated lung and no visible pleural effusion. (C) Control piglet with ascites (arrow), moderate dilation, mesocolonic edema, and hyperemia in the spiral colon. (D) VNA2-Tcd-treated piglet with moderate dilation and mild mesocolonic edema in the spiral colon. (E to I) Necropsy images of spiral colons from control and VNA2-Tcd-treated pigs inoculated with C. difficile spores. (E) Moderate mesocolonic edema, (arrow) hyperemia, focal mucosal ulceration, hemorrhages, and dilation in a control piglet. (F) Severe mesocolonic edema (arrow) in a control piglet. (G) Mild mesocolonic edema (arrow), dilation, and moderate hyperemia in a VNA2-Tcd-treated piglet. (H) Dilation and hyperemia in a VNA2-Tcd-treated piglet. (I) Dilation and hyperemia in an Ad/VNA2-Tcd-treated piglet. (J and K) Comparison of descending colons of control and VNA2-Tcd-treated piglets. Similar sections of the distal descending colon, approximately 6 cm in length, were collected from control and treated piglets at the time of necropsy. (J) Sections from control piglets demonstrating severe dilation, mesocolonic edema, multifocal hemorrhages, and thickening of the intestinal wall. (K) Sections from VNA2-Tcd-treated (purified or adenovirus expressing) piglets that show minimal or no dilation, no intestinal wall thickening, or hemorrhages.

Two groups of 5-day-old gnotobiotic piglets (12 piglets) were orally challenged with 10^6^ UK6 spores. The treatment group (3 piglets per treatment group and 6 untreated controls) was initially administered VNA2-Tcd (1 mg/kg) either 4 h prior to spore challenge (via i.p. delivery route) or 18 h postchallenge (via i.m. delivery route), followed by similar doses administered twice daily for the duration of the experiment. Three out of six control pigs were moribund (within 5 to 6 days post-spore inoculation) with signs of weakness, lethargy, severe (copious/continuous yellow or white mucoid or watery) diarrhea, and severe (red/bloody, externally visible, with thickening of the rectal wall) edematous rectal prolapse (Table 2). All pigs in the control and VNA2-Tcd-treated groups developed diarrhea within 48 h of inoculation with spores. In contrast, the signs of CDI disease were much less severe in the treated groups. Similar to mice and unlike the hamsters, none of the treated pigs became moribund. In addition, none developed rectal prolapse, and diarrhea was only mild to moderate in this group (Table 2). Half of the control piglets had pleural effusion and ascites (Fig. 5A and C). In contrast, pleural effusion and ascites were absent from VNA2-Tcd-treated piglets (Fig. 5B and D). Control piglets also had moderate to severe mesocolonic edema and dilation (Fig. 5C, E, and F) as well as hyperemia, mucosal ulceration, and hemorrhages (not shown). In contrast, treated piglets had mild to moderate mesocolonic edema with mild dilation (Fig. 5G through I) and moderate hyperemia (not shown).

**TABLE 2:** Pig clinical signs of disease

Treatment (no. of animals)	Gastrointestinal disease (%)^*a*^	Systemic disease (%)^*b*^	Fatal disease (%)^*c*^
Uninfected control (3)	Mild-moderate diarrhea (100), rectal prolapse (0)	0	0
UK6 spores + VNA2-Tcd (6)	Mild-moderate diarrhea (100), rectal prolapse (0)	0	0
UK6 spores + VNA2-Tcd-Adeno (9)	Mild-severe diarrhea (100), rectal prolapse (0)	0	0
UK6 spores + buffer (6)	Moderate-severe diarrhea (100), rectal prolapse (83)	50	50
UK6 spores + control-Adeno (6)	Moderate-severe diarrhea (100), rectal prolapse (50)	33	50

a Severity of gastrointestinal disease was determined by clinical signs of edema, hemorrhage, rectal prolapse, diarrhea, and gross and histopathologic lesions, ranging from mild to severe.

b Systemic signs include pleural effusion and ascites.

c Fatal disease indicates that piglets were euthanized due to the severity of the disease.

**FIG 6:**
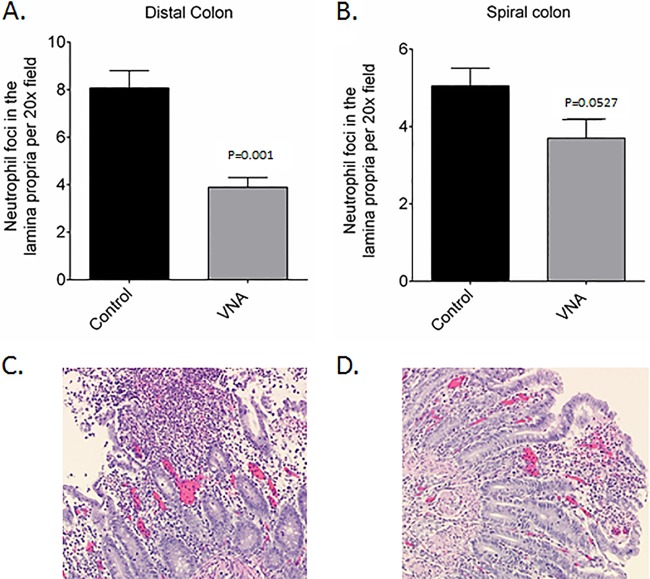
Evaluation of neutrophilic foci and histopathologic lesions in the colon and large intestine. (A and B) Quantitative evaluation of neutrophilic foci in distal colon (A) and spiral colon (B) of untreated control and VNA-treated piglets. (C) Untreated piglets with mucosal ulceration, hemorrhage, and marked neutrophilic infiltration and eruption of neutrophils and sloughed mucosa into the intestinal lumen. (D) Treated piglets with mild mucosal erosion and neutrophilic infiltration.

Microscopic examination of the large intestine identified submucosal edema as a cause of colonic mural thickening and mucosal neutrophilic infiltration as typical of CDI in piglets. As expected based on the clinical observations and gross findings, the main difference observed by light microscopy reflected lesion severity; more severe lesions were observed in the control versus those in the VNA-treated piglets. There was a trend for more severe submucosal edema in control piglets than in the VNA-treated piglets (not shown). Similarly, there were more neutrophils in the large intestines of the control piglets than in the VNA-treated piglets (Fig. 6A and B), which reached statistical significance for the distal colon (*P* = 0.001) but not for the spiral colon (*P* = 0.0527). Some control pigs had epithelial ulceration associated with neutrophilic colitis (Fig. 6C), while VNA-treated pigs did not develop ulceration associated with neutrophilic colitis (Fig. 6D).

Blood collected from pigs one time during the course of the experiment and at euthanasia determined that VNA2-Tcd serum levels ranged from 2.7 to 4.7 μg/ml (see Fig. S2C in the supplemental material). However, as observed in mice, serum neutralization abilities tested by cell cytotoxicity assay were similar for all treated pigs with a 1:2 dilution of serum providing about 50% reduction in cytotoxicity/cell rounding (Fig. S2D).

### Pig CDI challenge following gene therapy using adenovirus expressing VNA2-Tcd.

To further corroborate the efficacy of VNA2-Tcd in treating pigs for CDI, we employed a gene therapy approach in which an adenovirus vector (Ad/VNA2-Tcd) was administered that could promote the *in vivo* expression and secretion of VNA2-Tcd into the serum of treated pigs. A total of 15 gnotobiotic pigs were used to assess the effectiveness of Ad/VNA2-Tcd. Six control pigs were administered 1.0 × 10^11^ vp of adenovirus (i.v.) expressing an unrelated VNA, and 9 pigs were administered 1.0 × 10^11^ vp of Ad/VNA2-Tcd (i.v.). Each pig was treated twice with Ad/VNA2-Tcd, 1 day prior to oral inoculation with 10^6^ UK6 C. difficile spores and again 3 days after spore exposure. All six pigs in the control groups developed diarrhea within 48 h of inoculation with spores. Three of the six control pigs became moribund, all had moderate to severe diarrhea, and two had systemic signs of disease, including pleural effusion and ascites (Table 2). In contrast, none of the nine Ad/VNA2-Tcd-treated pigs became moribund, and diarrhea was predominantly mild to moderate with no other signs of disease (Table 2 and Fig. 5I and K). Microscopic examination again supported the trend that VNA treatment reduced lesion severity. More specifically, it was noted that treated pigs with high serum VNA levels had predominantly mild to moderate edema with two pigs (numbers 1 and 14), while pigs with low VNA levels showed marked edema (Table 3). Furthermore, Ad/VNA-treated pigs with the highest levels of serum VNA (numbers 6 and 13) had minimal or no edema (Table 3). Two of six control pigs (numbers 7 and 15) showed minimal to marked levels of edema (Table 3).

**TABLE 3:** Clostridium difficile infection and VNA2-Tcd levels in pig serum treated with adenovirus Ad/VNA2-Tcd^*c*^

Pig no.	Disease severity score	Ad/VNA-Tcd (ng/ml)	Mesocolon edema	Submucosa edema	Lamina propria edema	Neutrophilic colitis	Epithelium	Luminal contents
5	4	160	+/−	−	+/−	−	Intact	None
9	1	300	+	+/−	−	−	Intact	None
12	3	350	−	−	−	−	Intact	None
13	4	1,000	+	−	−	+/−	Intact	None
6	0	1,600	++	−	+/−	−	Intact	None
1	3	30	NA^*a*^	+++	++	++	Intact	Necrotic cell debris (+)
2	3	100	+	+	++	+/−	Intact	None
14	10	150	+	+++	++	++	Ulcerated	Necrotic cell debris (++)
7^*b*^	9	0	++	+/−	++	++	Intact	None
15^*b*^	11	0	++	+++	++	−	Intact	None

a NA, not available.

b These were control pigs that were treated with an unrelated VNA.

c −, none; +/−, mild to none; +, mild; ++, moderate; +++, severe.

**FIG 7:**
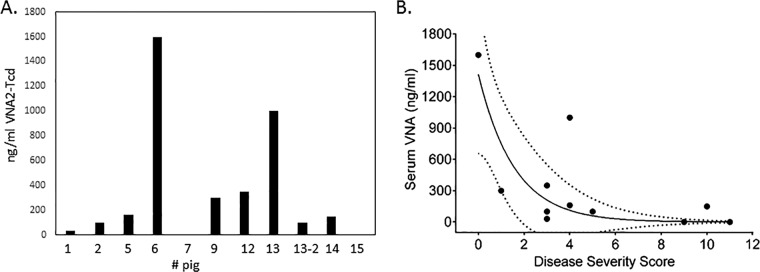
Ad/VNA2-Tcd detection in serum and disease correlation. (A) VNA2-Tcd levels detected in pig serum by ELISA using 0.5 μg/ml of TcdA or TcdB and serum diluted 1:10. Samples 7 and 15 correspond to control animals (unrelated Ad/VNA-treated piglets), and the remaining samples correspond to Ad/VNA2-Tcd-treated piglets. (B) CDI severity versus serum VNA concentration in pigs treated with Ad/VNA2-Tcd, assessed with Spearman's rank correlation (*r_S_* = −0.614; *P* = 0.04433). The dashed lines show the 95% confidence interval.

To determine whether there was a significant correlation between the severity of symptoms and the serum level of VNA2-Tcd, blood was collected from pigs one to three times during the course of the experiment and at euthanasia. Serum ELISAs showed that VNA2-Tcd serum levels ranged from 20 to 1,600 ng/ml at multiple time points during the experiment (Fig. 7A). The association between VNA concentration in pig serum and disease severity was analyzed using the Spearman rank correlation. This analysis demonstrated a significant negative relationship in which increased serum VNA concentration was clearly associated with lower CDI severity score (*r* = −0.614; *P* = 0.0443) (Fig. 7B).

## DISCUSSION

A single tetraspecific VNA protein comprised of four unique VHH binding agents, two each targeting C. difficile toxin A or toxin B at distinct neutralizing epitopes, reduced the severity of CDI clinical parameters and lesions in three different animal models: mice, hamsters, and gnotobiotic pigs. We expressed and purified VNA2-Tcd, tested the agent for *in vitro* toxin binding and neutralization activity, and then used the VNA to treat mice that received systemically delivered Tcd toxins. In the mouse model, a single dose of VNA (containing an albumin-binding peptide that improves VNA serum persistence in mice [60]) fully protected all groups that received the VNA against any signs or symptoms of toxemia, while all of the untreated mice died within 4 h of toxin administration. The same purified VNA2-Tcd also significantly protected mice, hamsters, and gnotobiotic piglets against systemic signs of disease in CDI challenge models. In these models, the toxins, likely leaking from intestinal lesions, enter the peritoneal space and bloodstream, inducing toxemia and systemic complications, such as ascites and pleural effusion (69, 70).

Since the VNA was administered systemically, we did not necessarily expect to see a significant protective effect on the integrity of the mucosa or the gastrointestinal (GI) tract; however, previous results indicated that there was a possibility that the VNA2-Tcd would be partially protective against GI pathology (56, 71). In mice and pigs, there was a significant protective effect against diarrhea, edema, and hemorrhage in addition to protection against systemic disease. VNA2-Tcd showed protection when administered i.p. against diarrhea in the mouse model (80% after the first dose and 100% after the second dose) and against edema and severe diarrhea in the piglet model. This suggests an ability of the agent to be effectively absorbed either through intestinal lesions (56, 71), loosening of epithelial cell tight junctions, or through normal portal absorption to some degree, thereby mitigating the effects of the toxin on microvillus degradation and neutrophil infiltration in the lamina propria and edema in the spiral and distal colon.

The difference seen in protection against GI disease between the mouse and piglet models of CDI may be attributed, at least in part, to the difference in the serum levels of VNA achieved by the different treatments. The mice received three doses of 1.25 to 2.5 mg/kg/day, while the hamsters and piglets received a dose of 1 to 2 mg/kg/day. Perhaps more important, the VNA contains an albumin-binding peptide that was selected for mouse albumin affinity (72). This peptide substantially increases the half-life of the VNA in mouse serum but has little or no apparent affinity for albumins from other species (60), such as pigs, and therefore, is unlikely to extend serum half-life. Increasing the dose of VNA administered to hamsters and piglets and/or employing a VNA with an albumin-binding peptide that binds hamster and pig albumin would likely improve its efficacy in these models to become closer to the high levels achieved in the mouse model.

Generally, the hamster model of CDI is extremely sensitive and requires as few as 100 spores to induce diarrhea (73). However, hamsters were reported to be relatively resistant to the UK6 strain of C. difficile (68). We did induce CDI disease in 100% of Syrian hamsters using 1,000 UK6 spores and found that any sign of diarrhea in hamsters was always fatal in our model. Hamsters sometimes died prior to external signs of diarrhea (wet tail) but with internal signs (lack of formed feces in the GI tract). Diarrhea in mice and pigs ranged in severity, and only severe diarrhea and systemic disease proved fatal in these animals. Since our treatment is designed to protect against the systemic effects of CDI, with only limited access to the GI tract, it is not surprising that this treatment was less effective in our hamster model where hamsters did not develop systemic disease and instead became moribund very soon after displaying symptoms of GI disease. Together, the clinical observations, necropsy, and microscopic findings suggest that morbidity in the hamster CDI model includes disease mechanisms that are independent from those resulting in edema and neutrophilic inflammation. Possibilities include electrolyte imbalances secondary to diarrhea and dehydration, hypovolemic shock due to fluid loss, and poor perfusion/reduced venous return to the heart secondary to compression of the caudal vena cava from massively dilated caeca.

Finding a method for the oral administration of the bioactive VNA may permit the more effective protection against diarrhea and edema seen in the hamster and piglet CDI treatment groups. Although VHHs are generally more stable to pH and temperature extremes than conventional antibodies (50), it seems likely that heteromultimeric VNAs will be susceptible to gastric enzyme degradation. To overcome this problem, VNAs may be lyophilized and delivered in drug capsules, using a nanoparticle delivery system, or by some form of gene therapy that promotes expression of VNA2-Tcd into the GI lumen (e.g., recombinant Lactococcus lactis) (74). Alternatively, an adenovirus that promotes secretion of VNA2-Tcd and that is engineered to selectively transduce intestinal epithelial cells (75, 76) may reduce intestinal edema and inflammation and the severity of diarrhea.

VNA2-Tcd is directed against the two secreted toxins, not against the bacteria, and does not involve the use of antibiotic therapy. Therefore, treatment with VNA2-Tcd is unlikely to promote the occurrence of relapse associated with antibiotic treatments against CDI, which is the major cause of fatal disease in humans (1). Previous results using therapeutic antibodies against CDI suggest that this is possible (35).

Using a single tetraspecific agent to express a polyprotein with multiple linked VHHs against two different Tcd toxins, and the feasibility of using microbial hosts for production, reduces manufacturing costs and reduces the complexity of clinical trials. The addition of an albumin-binding peptide that effectively binds human albumin would increase the serum persistence of the VNA, thereby increasing steady-state serum levels and reducing the frequency of doses required to effectively treat CDI pathology. The fact that VNAs can be effectively delivered by gene therapy (63–65) opens the possibility of developing single-dose therapies that provide prolonged efficacy and protect patients from relapses.

## Supplementary Material

Supplemental material
