# Supplementary material for: A Tetraspecific VHH-Based Neutralizing Antibody Modifies Disease Outcome in Three Animal Models of Clostridium difficile Infection
Source: Clin Vaccine Immunol. 2016 Sep 6;23(9):774–84. doi: 10.1128/CVI.00730-15 (PMC5014919; doi:10.1128/CVI.00730-15)
Supplement: Supplemental material [file supp_23_9_774__index.html]

A Tetraspecific VHH-Based Neutralizing Antibody Modifies Disease Outcome in Three Animal Models of Clostridium difficile Infection — Supplemental material 

# A Tetraspecific VHH-Based Neutralizing Antibody Modifies Disease Outcome in Three Animal Models of Clostridium difficile Infection

## Supplemental material

- Supplemental file 1 -

  Table S1. Mouse systemic toxin challenge. Table S2. *C. difficile* infection in hamsters. Fig. S1. VNA2-Tcd purification. Fig. S2. VNA2-Tcd detection and neutralizing activity in serum. Fig. S3. VNA2-Tcd detection in hamsters.

  PDF, 251K
