# Supplementary material for: A Tetraspecific VHH-Based Neutralizing Antibody Modifies Disease Outcome in Three Animal Models of Clostridium difficile Infection
Source: Clin Vaccine Immunol. 2016 Sep 6;23(9):774–84. doi: 10.1128/CVI.00730-15 (PMC5014919; doi:10.1128/CVI.00730-15)
Supplement: Supplemental material [file CVI.00730-15_zcd999095394so1.pdf]

Supplementary Table 1. Mouse systemic toxin challenge

Treatment Group				
Hours	Toxin A & Toxin B	Toxin A&B + VNA2-Tcd	VNA2-Tcd	Untreated
	# of surviving mice			
3	2/5	5/5	5/5	5/5
4	0/5	5/5	5/5	5/5
120	0/5	5/5	5/5	5/5

[a] hours post toxin challenge.
NA = not applicable

Supplementary Table 2. Clostridium difficile infection in hamsters

Hamster #	Submucosal Edema	Neutrophil Foci*
Control-2	marked	3
Control-6	mild	9
VNA2-Tcd-1	mild	4
VNA2-Tcd-2	mild	5
VNA2-Tcd-6	mild	13
VNA2-Tcd-8	marked	13
VNA2-Tcd-11	mild/none	1

*Neutrophil foci expanding the lamina propria or crypts were counted in 10 random from the most severely affected tissue sample, at 20x magnification.

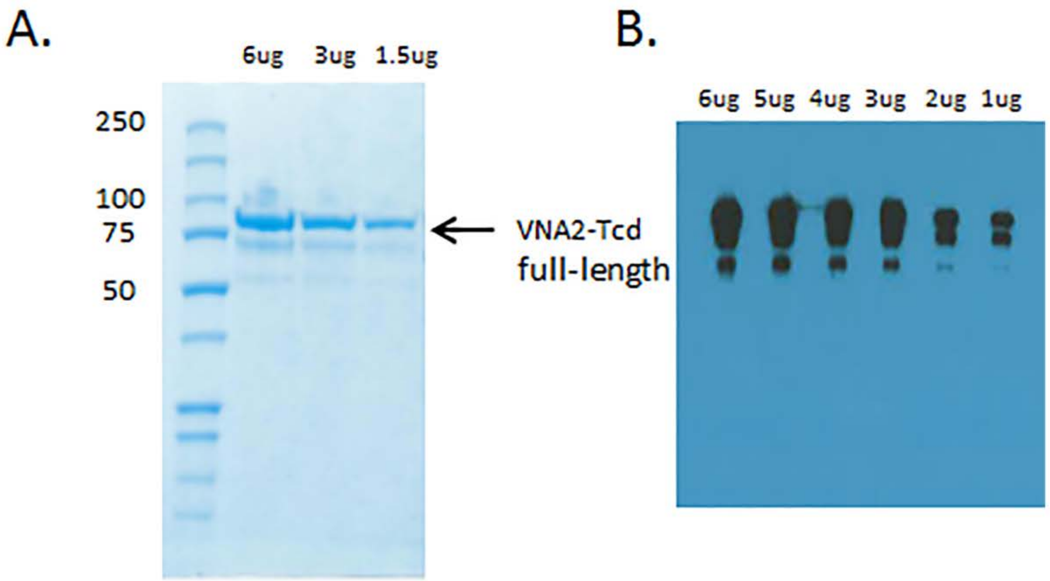

S1. Fig. VNA2-Tcd purification A) SDS-PAGE of VNA2-Tcd full-length heteromultimer purified by Ni-affinity followed by gel filtration chromatography. The top band in each well represents the full-length VNA2-Tcd heteromultimer. B) Western blot of serially diluted VNA2-Tcd using anti-E tag antibody to detect VNA full length, trimer and dimer VNA products. Loading amounts are indicated above each lane.

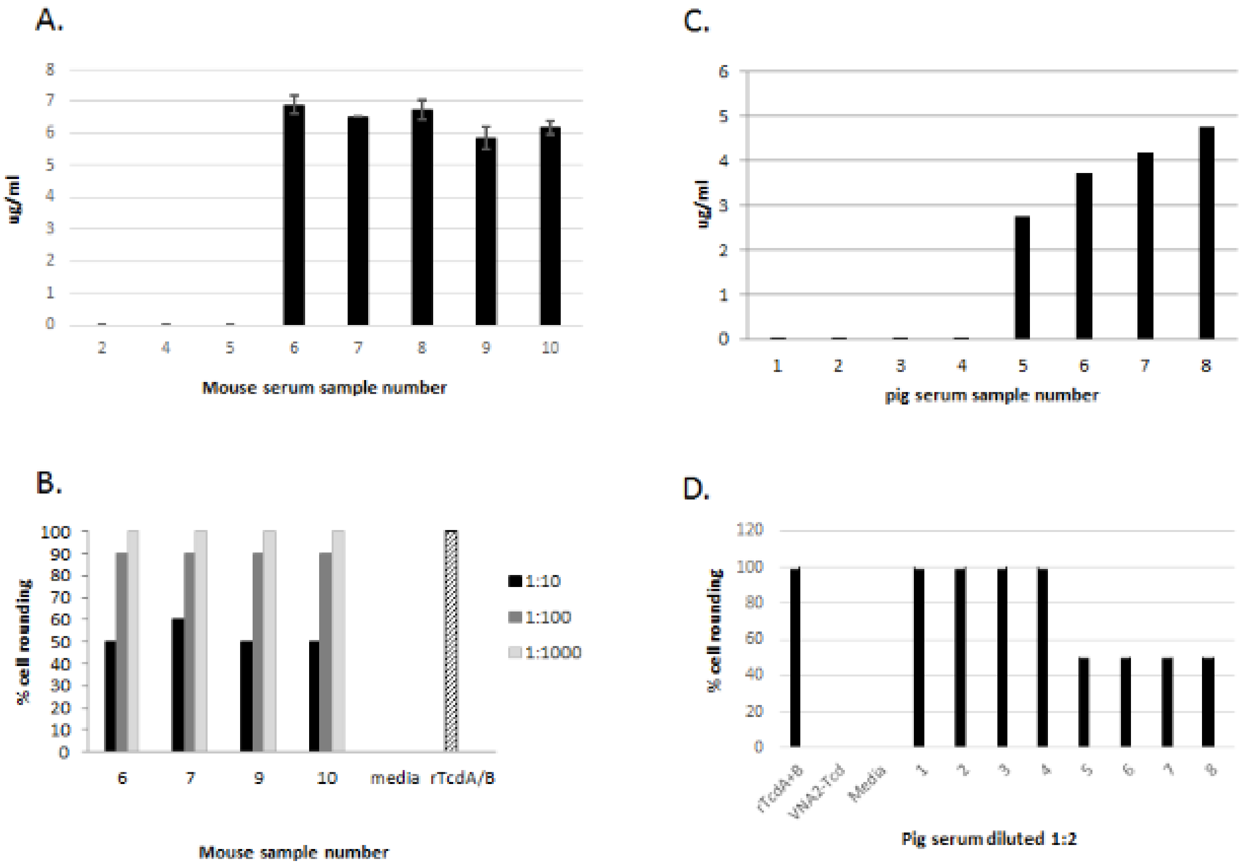

S2 Fig. VNA2-Tcd detection and neutralizing activity in serum. A) VNA2-Tcd levels were assessed by ELISA. Samples 2, 4, and 5 correspond to control (untreated mice) and 6-10 correspond to VNA2-Tcd treated mice. A standard curve was generated using serially diluted purified VNA2-Tcd. B) In vitro neutralization assay using Vero cells incubated with rTcdA (19.2 ng/ml) and rTcdB (1.8 ng/ml) per well for 24hrs and 1:10 (black bar), 1:100 (dark gray bar), 1:1000 (light gray bar) dilutions of mouse serum. Percent cell rounding (cytotoxicity) was assessed after 24 hours. rTcdA and rTcdB together shows 100% cell rounding, media alone shows 0% cell rounding, while rTcdA/B + mouse serum shows 50% cell rounding for 1:10 dilutions, 90% cell rounding for 1:100 dilutions and 100% cell rounding for 1:1000 dilutions. C) VNA2-Tcd levels detected in pig serum using the same method. Samples 1-4 correspond to

control (untreated piglets) and samples 5-8 to VNA2-Tcd treated piglets. D) In vitro neutralization assay using vero cells incubated with rTcdA (2ng/ml) and rTcdB (0.25 ng/ml) per well for 24hrs and 1:2 dilutions of control (untreated) piglet serum and VNA2-Tcd treated piglets. rTcdA and rTcdB together shows 100% cell rounding, media alone and VNA2-Tcd alone shows 0% cell rounding, rTcdA/B + control piglet serum shows 100% cell rounding (samples 1-4), and rTcdA/B + control piglet serum shows 50% cell rounding (samples 5-8).

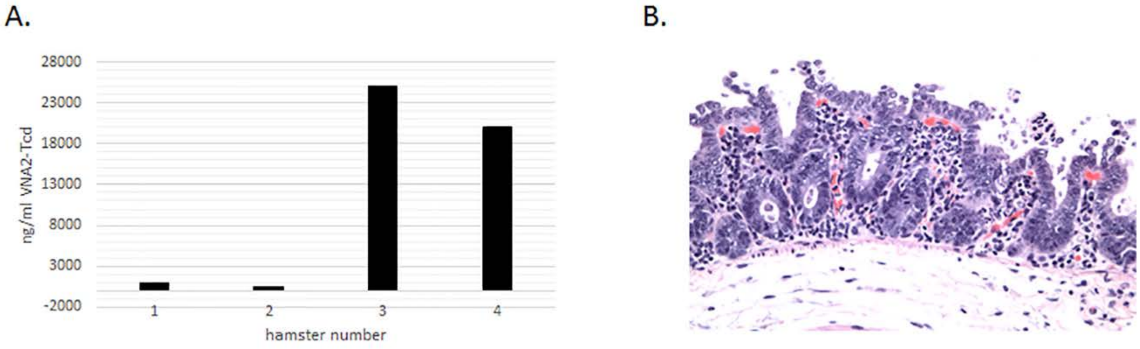

S3 Fig. VNA2-Tcd detection in hamsters. A) VNA2-Tcd levels detected in hamster serum by ELISA using 0.5µg/ml of rTcdA or rTcdB and serum diluted 1:10. B) A 20x magnification from control hamster showing an example of neutrophil focus in the lamina propria of the large intestine.
